# Virus Infection of *Aspergillus fumigatus* Compromises the Fungus in Intermicrobial Competition

**DOI:** 10.3390/v13040686

**Published:** 2021-04-16

**Authors:** Hasan Nazik, Ioly Kotta-Loizou, Gabriele Sass, Robert H. A. Coutts, David A. Stevens

**Affiliations:** 1 California Institute for Medical Research, 2260 Clove Dr., San Jose, CA 95128, USA; hasannazik01@gmail.com (H.N.); gabriele.sass@cimr.org (G.S.); 2 Department of Life Sciences, Imperial College London, London SW7 2AZ, UK; i.kotta-loizou13@imperial.ac.uk; 3 Department of Clinical, Pharmaceutical and Biological Sciences, University of Hertfordshire, Hatfield AL10 9AB, UK; r.coutts@herts.ac.uk; 4 Division of Infectious Diseases and Geographic Medicine, Stanford University School of Medicine, Stanford, CA 95128, USA

**Keywords:** *Aspergillus*, fungal virus, *Pseudomonas*, intermicrobial competition

## Abstract

*Aspergillus* and *Pseudomonas* compete in nature, and are the commonest bacterial and fungal pathogens in some clinical settings, such as the cystic fibrosis lung. Virus infections of fungi occur naturally. Effects on fungal physiology need delineation. A common reference *Aspergillus fumigatus* strain, long studied in two (of many) laboratories, was found infected with the AfuPmV-1 virus. One isolate was cured of virus, producing a virus-free strain. Virus from the infected strain was purified and used to re-infect three subcultures of the virus-free fungus, producing six fungal strains, otherwise isogenic. They were studied in intermicrobial competition with *Pseudomonas* *aeruginosa. Pseudomonas* culture filtrates inhibited forming or preformed *Aspergillus* biofilm from infected strains to a greater extent, also seen when *Pseudomonas* volatiles were assayed on *Aspergillus*. Purified iron-chelating *Pseudomonas* molecules, known inhibitors of *Aspergillus* biofilm, reproduced these differences. Iron, a stimulus of *Aspergillus*, enhanced the virus-free fungus, compared to infected. All infected fungal strains behaved similarly in assays. We show an important consequence of virus infection, a weakening in intermicrobial competition. Viral infection may affect the outcome of bacterial–fungal competition in nature and patients. We suggest that this occurs via alteration in fungal stress responses, the mechanism best delineated here is a result of virus-induced altered *Aspergillus* iron metabolism.

## 1. Introduction

Viruses naturally infecting *Aspergillus* wild-type strains have been known since 1970 [1]. These viruses have double-stranded (ds) or single-stranded (ss) RNA genomes and may be conventionally encapsidated. They do not have an extracellular phase in their replication cycle but can be transmitted horizontally via hyphal fusion and vertically via spore production. However, little is known about how they affect *Aspergillus* physiology or virulence.

The virus family *Polymycoviridae* was initially reported in 2015 [2] and was officially recognized by the International Committee on Taxonomy of Viruses in 2020 (https://talk.ictvonline.org/; accessed on 5 April 2020). *Polymycoviridae* currently accommodate one genus *Polymycovirus* and 10 species, including *Aspergillus fumigatus polymycovirus 1*. Members of the *Polymycoviridae* family and related viruses have usually four [2,3] and up to eleven [4] dsRNA genomic segments. A majority of polymycoviruses are not conventionally encapsidated [2,3], although filamentous particles have been reported in one case [5] and are infectious as dsRNA [2,5].

There has been extensive research in many laboratories, for decades, regarding intermicrobial interactions, particularly *Pseudomonas–Aspergillus* interactions (recently reviewed [6,7,8,9,10,11,12,13]), two microbes that coexist, and likely compete, in nature and in patients, particularly persons with cystic fibrosis and immunocompromised hosts. A central facet of the competition is the mutual battle to withhold iron from the competitor. The *Pseudomonas* siderophore and pyoverdin are its principal weapons used against *Aspergillus* in iron-restricted liquid environments [14], along with the Pseudomonas Quinolone Signal (PQS) [15] and pyocyanin, which is likely a more direct toxin in iron-rich environments [16]. *Aspergillus* counters the battle for iron with its own siderophores [17]. In a non-liquid air environment, *Pseudomonas* can also generate volatiles that can inhibit *Aspergillus* [18]. Presently we studied one aspect of the possible effect of virus infection of *Aspergillus fumigatus* on this intermicrobial interaction, using tools that have previously been quantitatively standardized.

AF293 is a very common *A. fumigatus* laboratory strain, and its genome has been fully sequenced [19]. Two versions, one in the USA and one in the UK, separated by at least 10 years, were studied, and both were found to carry the same virus, Aspergillus fumigatus polymycovirus 1 (AfuPmV-1) [2]. The UK strain was cured of AfuPmV-1, resulting in virus-free and virus-infected isogenic lines as previously described [2]. To control for differences found between infected and uninfected strains, the virus-cleared strain was re-infected in three experiments with the same virus that was present in the original strain, producing three re-infected strains, as previously described [2].

## 2. Materials and Methods

Isolates: The USA and UK AF293 strains were designated by the California Institute for Medical Research (CIMR) # 10-53 and 18-95, respectively. *A. fumigatus* AF293 strain (18-95) was cured from AfuPmV-1, using the protein synthesis inhibitor cycloheximide [2], producing a strain now designated 18-42. AfuPmV-1 was purified by differential polyethylene glycol precipitation and ultracentrifugation [2]. Purified AfuPmV-1 was re-introduced in the virus-free *Aspergillus* by protoplast transfection [2]. This was done 3 times, producing infected strains, designated CIMR 19-40 1A, 19-41 2A, and 19-42 3A. The presence or absence of AfuPmV-1 was confirmed by dsRNA extraction, Northern blotting, and RT-qPCR as previously described [2] (Figure S1).

PAO1 and PA14 are extensively studied widely distributed laboratory reference *P. aeruginosa* strains [16]. The use of all microbes in the CIMR laboratory is approved by the CIMR Biological Use Committee (approval no. 001-03Yr.14, 001-05 Yr.1).

In brief, methods used for generating and testing *Pseudomonas aeruginosa* supernatants and molecules on *Aspergillus* biofilms, as well as sources for materials, were as previously described [14,15,16,17,20].

Materials: Pyoverdin, PQS (Pseudomonas Quinolone Signal, 2-heptyl-3-hydroxy-4(1*H*)-quinolone), XTT (2,3-bis(2-methoxy-4-nitro-5-sulfophenyl)-2H-tetrazolium-5-carboxanilide inner salt), menadione, iron (FeCl_3_), and RPMI 1640 medium were purchased from Sigma-Aldrich (St. Louis, MO, USA). Trypticase soy agar (TSA) was purchased (Lonza, Walkersville, MD, USA) and prepared per manufacturer instructions. RPMI 1640 agar was prepared: Briefly, 7.5 g Bacto Agar (Carolina Biological Supply Co., Burlington, NC, USA) in 100 mL distilled water was autoclaved and mixed with 400 mL pre-warmed RPMI-1640 medium.

Methods: *P. aeruginosa* supernatants were prepared as detailed previously [20]. Briefly, *P. aeruginosa* (5 × 10^7^ cells/mL) was incubated in RPMI 1640 medium with or without addition of FeCl_3_ at 37 °C and 100 rpm for 24 h. Bacterial growth was measured at 600 nm, using a spectrophotometer (Genesys 20, Thermo Fisher Scientific Inc., Waltham, MA, USA). Bacterial cultures were centrifuged at 200× *g* for 30 min, at room temperature, and filtered for sterility (0.22 micron).

Plate assays for the determination of *Pseudomonas* effects on *Aspergillus* forming or preformed biofilms forming biofilm assay: *A. fumigatus* conidia (2.5 × 10^4^/mL) was seeded in test substances in RPMI 1640 medium, in 96-well plates, at 37 °C, overnight (16 h). Preformed biofilm assay: *A. fumigatus* conidia (2.5 × 10^4^/mL) was seeded in RPMI 1640 medium, in 96-well plates, at 37 °C, for 24 h. The plates were washed with phosphate-buffered saline, and test substances were added. Plates were incubated at 37 °C, overnight (16 h).

All assays were evaluated by XTT metabolic assay [20,21]. Briefly, 150 microliters of an XTT-menadione mixture was added to each test well and incubated at 37 °C, for one hour. Supernatants from each well (100 microliters) were assayed, using a plate reader (Vmax, Molecular Devices, San Jose, CA, USA) at 490 nm.

Assays for testing inhibition of planktonic *Aspergillus* growth, by *Pseudomonas* supernatants or antifungal drugs, were as described [22]; Minimum Fungicidal Concentration (MFC) was defined as ≥96% killing of the inoculum [23].

As in the manner of previous studies [18], the effect of *Pseudomonas* volatiles on *Aspergillus* growth was assessed by cutting away a 3 mm–wide strip, with a scalpel, out of the agar of an 8 cm diameter TSA agar plate, along a diameter, to separate the plate into 2 semicircular noncontiguous agar halves. The halves were inoculated with 10 microliters of a 10^7^/mL suspension, in RPMI1640 of *Pseudomonas*, or of *Aspergillus* conidia, and this co-culture incubated for 72 h, at 37 °C, and then the *Aspergillus* colony area (πr^2^) was measured. Each experiment involved 3 replicates.

Statistical analysis: Results were analyzed with Student’s *t*-test if two groups were compared, and one-way analysis of variance (ANOVA) combined with Tukey’s post-test for multiple comparisons. All data are expressed as the mean and standard deviation. Data reported as percentages of the control value were compared with Student’s *t*-test after arcsin transformation of the proportions; these data are presented as percentages. Assays used 4–8 replicate wells for each group studied, for statistical purposes.

## 3. Results

Effect of virus infection on *A. fumigatus* physiology in the context of the assays to be performed: To assist in interpretation of possible *P. aeruginosa* effects on *A. fumigatus* in the assays selected to test such effects, we needed to assess *A. fumigatus* function in the absence of *P. aeruginosa*, which is the *A. fumigatus* control we would use for assessing *P. aeruginosa* effects. Seven experiments assessing *A. fumigatus* biofilm metabolism were performed in the milieu to be used for later testing *P. aeruginosa* effects: RPMI 1640, in the plates, temperature, etc., described. There were 5 to 17 replicates for each of the three *A. fumigatus* strains (Af 18-42, virus-free, and isogenic controls, infected, 10-52 and 18-95) under study, in each of the seven experiments (a total of 212 replicates). The mean ± SD for the XTT assays, A_490_, for the seven experiments was 0.359 ± 0.12, 0.387 ± 0.13, and 0.455 ± 0.14, for the three isolates, respectively. None of these is significantly different from the others, *p* > 0.05.

Aside from metabolism (assessed by XTT), the other assay of *P. aeruginosa* effects (below) involved effects of volatiles on *A. fumigatus* growth on TSA agar. As will be reiterated, there were, again, not any differences found in the *A. fumigatus* controls (absence of *P. aeruginosa*) under the same culture conditions, as described for those assays.

**Figure 1:**
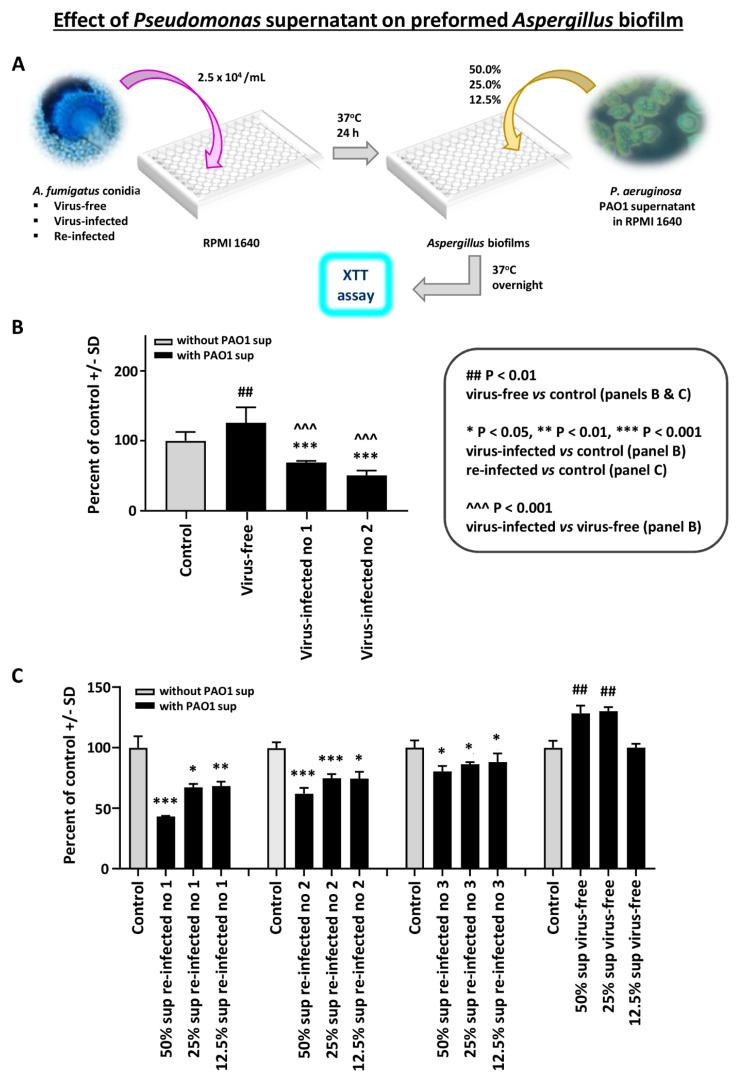
Effect of *Pseudomonas* planktonic supernatant on preformed *Aspergillus* biofilm. We studied preformed *Aspergillus* biofilm (i.e., 16–40 h culture) and *Pseudomonas* (PAO1) supernatants. The read-out is the XTT assay, to quantitate *Aspergillus* metabolism. Data are presented as % of control (medium alone). (**A**). Diagram of methods. (**B**). We see the virus-free preformed biofilm (left dark bar) is resistant to *Pseudomonas* supernatant. In contrast, the 2 infected strains (10-53 no. 1; 18-95 no. 2) are significantly inhibited. The virus-free *Aspergillus* strain even appears to be stimulated by the *Pseudomonas* supernatant. (**C**). This shows a dose titration of the *Pseudomonas* supernatant (50% to 12.5%) effect, comparing the virus-free *Aspergillus* (18-95) to that same strain, re-infected. The 3 re-infected *Aspergillus* strains (19-40 1A, 19-41 2A, and 19-42 3A) are re-infected nos. 1, 2, and 3, respectively. The 4 bars, black and gray, at the right are the virus-free *Aspergillus*, and the stimulatory effect of *Pseudomonas* supernatant is again seen on the virus-free strain, at the lower dilutions. The re-infected strains (all the remaining quartets of bars) are inhibited by *Pseudomonas* supernatant. All *Aspergillus* strains show a dose-response.

Effect of *Pseudomonas* planktonic supernatant on preformed *Aspergillus* biofilm: In the first study with preformed *Aspergillus* biofilm (i.e., after 16 h, till 40 h culture), and *P. aeruginosa* PAO1 planktonic supernatants, we found the virus-free preformed biofilm resistant to *Pseudomonas* supernatant (Figure 1B). In contrast, the two infected strains were significantly inhibited.

In a dose titration of *Pseudomonas* supernatant (Figure 1B), we compared the virus-free *Aspergillus* to the three re-infected *Aspergillus* strains. The re-infected strains were inhibited by *Pseudomonas* supernatant. These observations were confirmed in two additional experiments.

**Figure 2:**
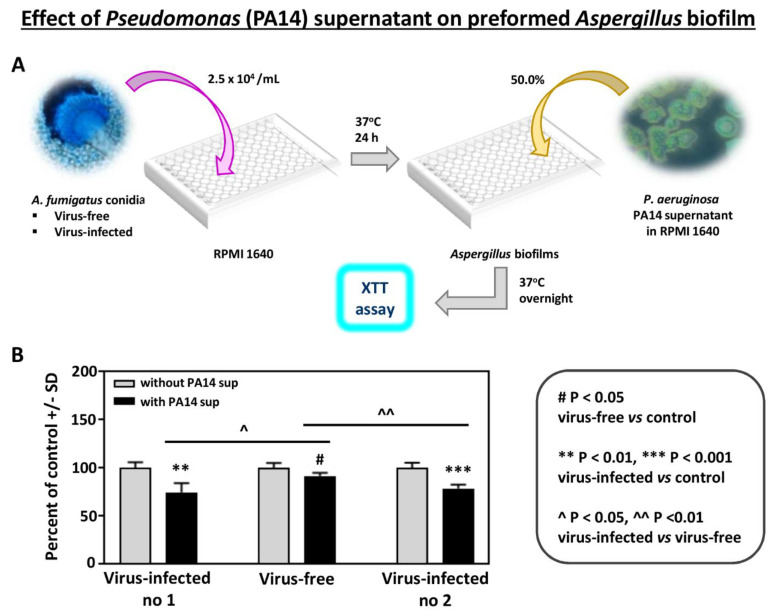
Effect of *Pseudomonas* (PA14) planktonic supernatant on preformed Aspergillus biofilm. (**A**). Diagram of methods. (**B**). All 3 *Aspergillus* strains are inhibited (XTT assay) at a 1:2 dilution of *Pseudomonas* supernatant (black bars), comparing to their own controls (medium alone)). The virus-free *Aspergillus* is less inhibited compared to the infected isolates, compared to 10-53 (left), and to 18-95 (right).

In addition, to study the effects of another *Pseudomonas* strain, we compared the effect of supernatant of *Pseudomonas* strain PA14 on preformed *Aspergillus* biofilm (Figure 2). As with PAO1, the effects were significantly stronger on the infected strains.

**Figure 3:**
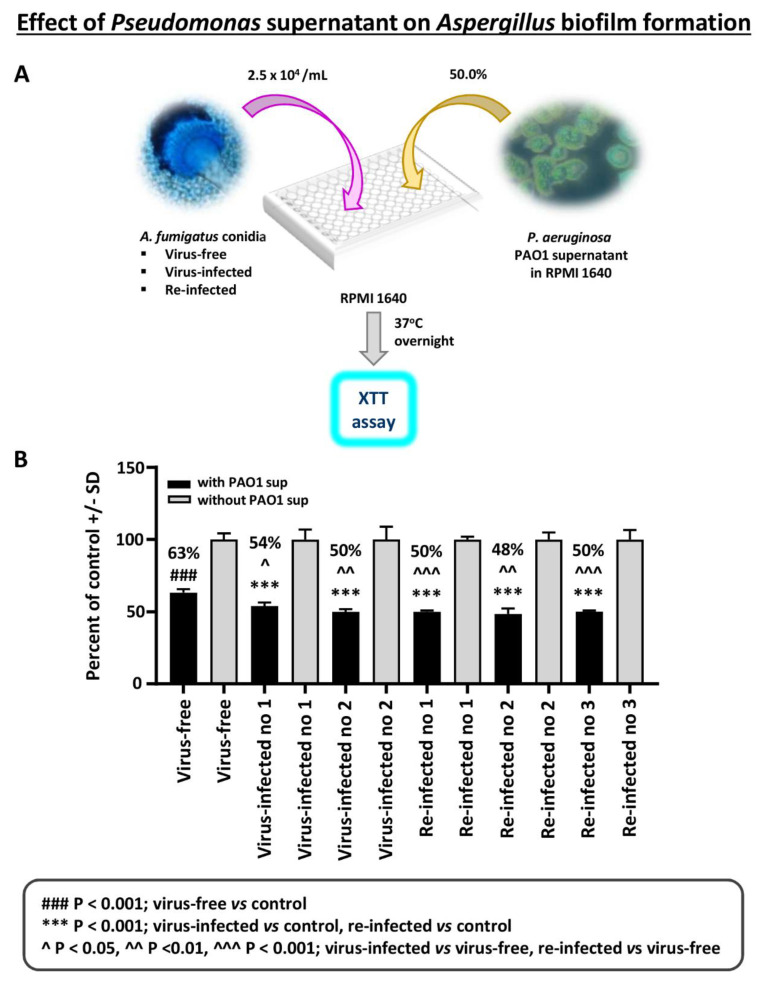
Effect of *Pseudomonas* planktonic supernatant on AF293-virus free or AF293-infected biofilm formation. (**A**). Diagram of methods. (**B**). Black bars are the result of treatment; gray bars are the control (medium alone) value, set at 100%. The 2 bars at the left are the virus-free *Aspergillus*. “Infected nos. 1 and 2” are the 2 infected strains, 18-95 and 10-53, respectively; and the remainder the re-infected strains, as indicated (19-40 1A, 19-41 2A, and 19-42 3A are re-infected nos. 1, 2, and 3, respectively). All isolates are inhibited by a 1:2 dilution of *Pseudomonas* PAO1 supernatant; the % inhibition compared to their controls is indicated above the black bars. The virus-free *Aspergillus* is significantly less inhibited, comparing the virus-free to the other isolates.

Effect of *Pseudomonas* planktonic supernatant on AF293-virus free or AF293-infected biofilm formation: The effect of planktonic *Pseudomonas* (PAO1) supernatant on biofilm formation (i.e., initial 16 h of culture) by *A. fumigatus* was studied (Figure 3). All isolates are inhibited by *Pseudomonas* supernatant. The virus-free *Aspergillus* is significantly inhibited less.

A second experiment of this type was performed with PA14 supernatant. PA14 supernatants proved to be much more inhibitory in these experiments with biofilm formation than PAO1 supernatants, consistent with the reported greater virulence of PA14 compared to PAO1 [24], owing to a mutation. At a 1:100 dilution, the two infected and the virus-free strains were all greatly inhibited, to <25% of controls (*p* < 0.001). At a 1:500 dilution, all three strains were inhibited ≤50% of controls, but the virus-free was inhibited significantly less than either two infected strains (*p* < 0.05 compared to 18-95, and *p* < 0.001 compared to 10-53; the two infected strains were not significantly different from each other). At a 1:1000 dilution, none of the three *Aspergillus* strains was inhibited.

**Figure 4:**
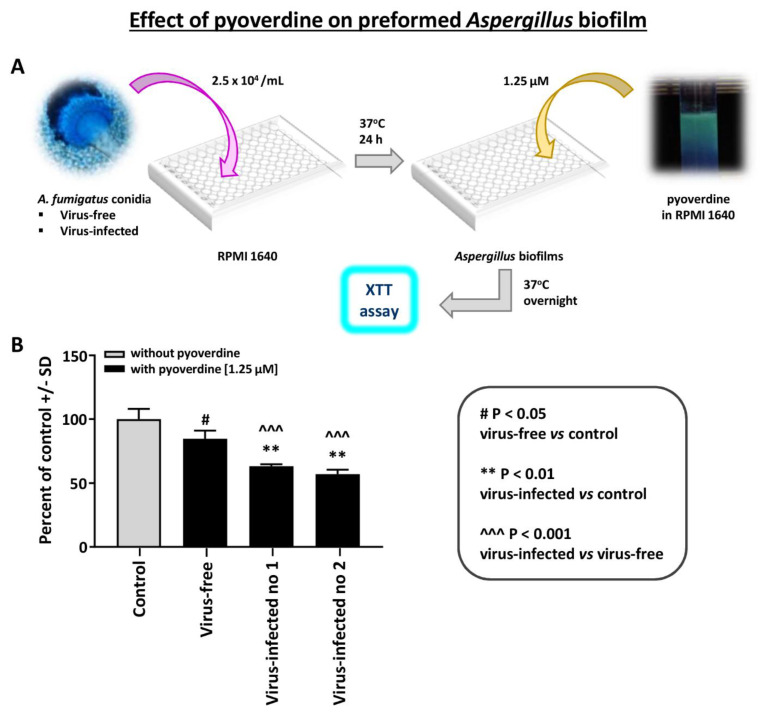
Effect of pyoverdine on *Aspergillus* strains. This figure compares the effect of pyoverdine, 1.25 micromolar, on preformed *Aspergillus* biofilm (XTT assay). (**A**). Diagram of methods. (**B**). The virus-free *Aspergillus* (black bar, second bar from left) is significantly less inhibited. “Infected nos. 1 and 2” are the two infected strains, 18-95 and 10-53, respectively.

The effect of pyoverdine on *Aspergillus* strain pyoverdine is a *Pseudomonas* siderophores that is inhibitory to *Aspergillus* [14]. We compared the effect of pyoverdine on preformed *Aspergillus* biofilm (XTT assay) (Figure 4). The virus-free *Aspergillus* was significantly less inhibited by pyoverdine. A similar difference was noted with 12.5 micromolar pyoverdine (not shown); the two infected *Aspergillus* strains were inhibited more at this higher concentration, and *p* < 0.001 compared to the virus-free.

**Figure 5:**
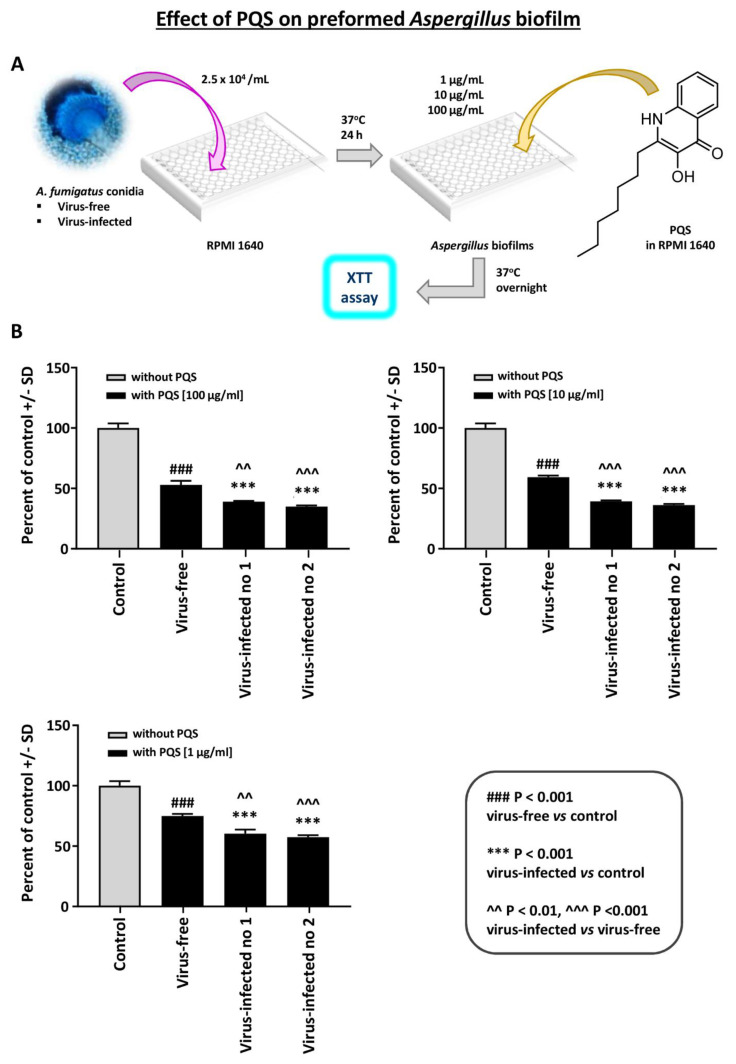
Effect of Pseudomonas Quinolone Signal (PQS) on preformed *Aspergillus* biofilm. This figure shows a dose-titration of 100, 10, and 1 mcg/mL PQS on preformed *Aspergillus* biofilm metabolism. (**A**). Diagram of methods. (**B**). At every concentration, the 2 virus-infected strains (“Infected nos. 1 and 2” are 18-95 and 10-53, respectively) are significantly more inhibited than the virus-free, which is the black bar, second bar from the left, of each segment. Control, RPMI 1640 medium with ethanol concentrations equivalent to that in PQS test reagent.

Effect of PQS on preformed *Aspergillus* biofilm: PQS is an important *Pseudomonas* exoproduct that is involved in quorum sensing and the production of virulence factors, and it also has iron-binding properties. It inhibits *Aspergillus* [15] under iron-restricted conditions (e.g., RPMI1640 medium). We performed a dose-titration of PQS effect on preformed *Aspergillus* biofilm metabolism (Figure 5). At every concentration, the two virus-infected strains were significantly more inhibited than the virus-free.

Effect of *Pseudomonas* volatiles on *Aspergillus* strains with the assay method and media described (see Methods): In studies with many *Pseudomonas* laboratory strains and clinical isolates, and several *Aspergillus* isolates, we find *Pseudomonas* volatiles to be inhibitory to *Aspergillus* [18]. The entities responsible appear to be small lipophilic organic molecules.

**Figure 6:**
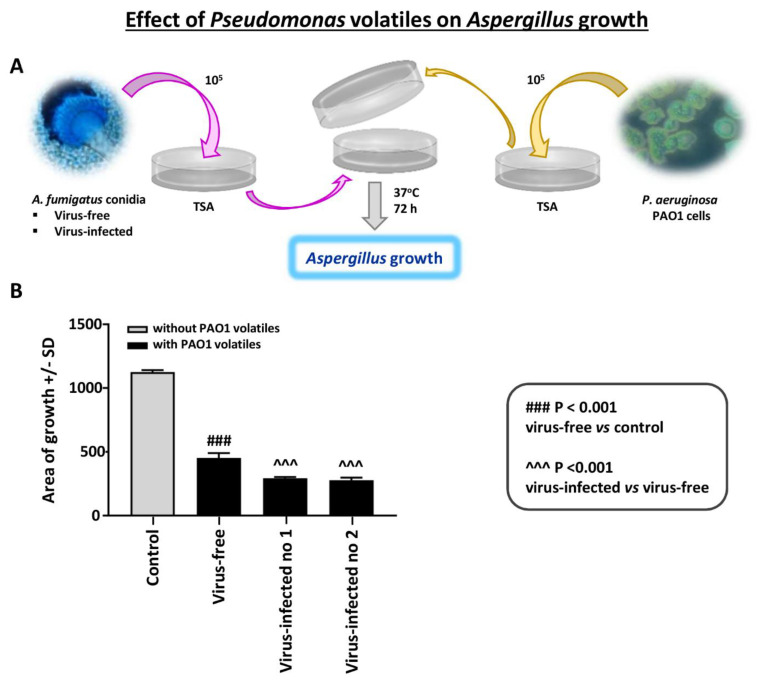
Effect of *Pseudomonas* volatiles on *Aspergillus* strains. An *Aspergillus* isolate was inoculated on trypticase soy agar (TSA) agar and sealed together with an inverted plate with *Pseudomonas* PAO1 growing on it; there was no direct contact between the plates. There were 4 plates prepared for each *Aspergillus–Pseudomonas* combination; means and SD are shown. The control is the same 2-plate apparatus, with no *Pseudomonas* on the inverted plate; two plates/*Aspergillus* isolate. (**A**). Diagram of methods. (**B**). The 3 *Aspergillus* isolates’ growth in the absence of *Pseudomonas* were not different (colony area) on TSA; thus, these growth results are merged and shown as “Control”. After ≥3 days of incubation, the virus-free *Aspergillus* (left black bar) was inhibited significantly less than the infected (“Infected nos. 1 and 2” are 10-53 and 18-95, respectively). The areas of growth here were measured after 72 h of co-exposure; prior to that, there were no statistically significant differences, as all the *Aspergillus* colonies continued to slowly enlarge over time.

We here tested *Aspergillus* growth on agar in the presence of *Pseudomonas* growth on the same plate, as described. In the absence of *Pseudomonas*, the virus-free and the two virus-infected strains grew equally well on TSA agar. We found that the virus-free *Aspergillus* was inhibited significantly less by *Pseudomonas* than the infected (Figure 6). The experiment shown is representative of three experiments with identical results.

**Figure 7:**
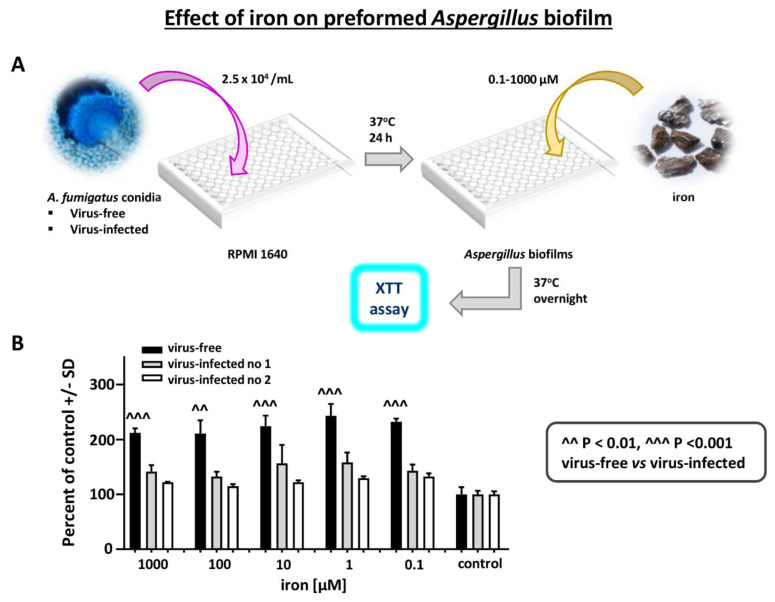
Effect of iron on *Aspergillus* strains. Differential effect of FeCl_3_ on virus-free compared to virus-infected *Aspergillus* preformed biofilm, XTT assay, over a range of iron concentrations. (**A**). Diagram of methods. (**B**). The 3 bars at the right are the control in RPMI1640 without added iron. Within each triplet, from left to right, are the virus-free *Aspergillus* (the black bars), and flanking the black bar each time, the infected *Aspergillus* strains (“infected nos. 1 and 2” are 10-53 and 18-95, respectively). Iron stimulates the virus-free strain to a significantly greater extent, at all iron concentrations.

Effect of iron on *Aspergillus* strains: Iron stimulates *Aspergillus* biofilm growth [25]. We examined a possible differential effect of FeCl_3_ on virus-free vs. virus-infected *Aspergillus* preformed biofilm metabolism (XTT assay), over a range of iron concentrations (Figure 7). Iron stimulated the virus-free strain to a significantly greater extent, at all iron concentrations.

Effect of PAO1 planktonic filtrate on AF293-virus free or AF293-infected planktonic growth: The differential effect of *Pseudomonas* on planktonic *Aspergillus* growth was marginal (Supplementary Text and Figure S2), compared to the effects described above (Figure 1 and Figure 2) on *Aspergillus* biofilm.

Drug susceptibility: We compared the susceptibility of virus-free vs. virus-infected *Aspergillus* strains to amphotericin B, voriconazole, and caspofungin and found no differences. The MICs and MFCs (mcg/mL) for the virus-free and virus-infected *Aspergillus* strains were, for amphotericin B, 1 and >8, respectively; for caspofungin, 12.5 and >50; and for voriconazole, ≤0.5 and >8.

## 4. Discussion

Fungal viruses have been described in 22 viral taxa, with five in the genus *Aspergillus,* including members of the established families *Chrysoviridae*, *Narnaviridae*, *Partitiviridae*, *Polymycoviridae*, and *Totiviridae* [26]. It has been estimated that 30–80% of fungal species are infected, and >100 fungal species [27,28]. Most mycoviruses are dsRNA, un-enveloped; ssRNA viruses appear to be increasingly common [26,29,30]. They are less common in teleomorphs [26]. Most are transmitted by cell-to-cell transmission, and most are vertically transmitted via conidiospores [26,30,31]. Extracellular transmission [32] and transmission via mycophagous insects have been described [33] for the only DNA mycovirus known to date [34]. Most mycoviruses do not appear to integrate in the host genome, with the exception of families *Metaviridae* and *Pseudoviridae*. The viruses tend to be latent, persist, and are difficult to eliminate [26,30]. Multiple different virus infections in a fungus are common [27], for example, the *A. foetidus* mycovirus complex [35,36,37]. Whereas the majority of mycoviruses appears to cause no obvious phenotypic changes, nor a debilitating effect on the host fungus, some mycoviruses are lytic, and some have been described that can cause plaques in fungal lawns in vitro, and the dsRNA has been implicated [27,29,38]. Treatment with an antifungal is one stimulus described that can trigger a lytic virus in *Candida albicans* [38]. Fungal antiviral defense has been associated with RNA silencing [26,28].

All known mycovirus genomes encode for replication enzymes, such as RNA-dependent RNA polymerase. Conversely, not all encode capsid polypeptides or are enclosed in traditional, spherical, or filamentous, protein capsids. This is the case for polymycoviruses and related viruses [2,4], that can produce infections with only naked dsRNA [2,5,31]. In some yeasts, such as *Saccharomyces cerevisiae*, mycoviruses encode protein toxins that kill other fungi, in some by inhibiting glucan synthesis [29,39] or DNA synthesis [40]. Some fungal viruses trigger or suppress production of fungal toxins harmful to mammalian species [31], such as aflatoxin in *A. flavus* [1]. Fungal phytopathogens have been associated preferentially with virus-free strains [27], and virus-induced hypovirulence has been utilized in the field against *Cryphonectria parasitica*, the fungus causing chestnut blight [28]. There has been interest in these viruses, as to what the effects on mammalian hosts might be, because of the known immunological effects of dsRNA [29].

In *Aspergillus*, ~10–50% of isolates in a species have been described as infected [26]. Seven to 19% of *A. fumigatus* clinical isolates are virus-infected [41,42]. The viruses do not appear to integrate in the host genome. Infection even across fungal genera has been described in this group of viruses [1], and some *Aspergillus* viruses can produce infections with only naked dsRNA [2,5,31].

Whereas the preponderance of evidence associates non-latent virus infection with fungal hypovirulence, as is the case for a variant of AfuPmV-1 that has one additional dsRNA element and was tested in a murine model [43], there is evidence in *Aspergillus* that some viruses may increase virulence [1,31,44], or have no effect on virulence [1,44,45]. Other studies have indicated that viruses in two *Aspergillus* viral taxa have no effect on antifungal drug susceptibility [45], as was the observation in our study.

In our study, we showed that an *Aspergillus* virus alters *A. fumigatus* phenotype. There is evidence for an active competition between *Aspergillus* and *Pseudomonas*, a competition that may occur in soil and water. As these two microbes are the most common fungal and bacterial pathogens in many patient groups, the outcome of the competition could be very important for the long-term welfare in the patients (summarized in References [6,7,8,9,10,11,12,13]). We have studied here well-documented *Pseudomonas* weaponry against *Aspergillus* in biofilm form, a competition that is likely most important to certain microbial residences, such as in the lung, in immunocompromised hosts, and also, particularly, in persons with cystic fibrosis. The studies employed well-described assays, in defined media, that have previously quantitated *Pseudomonas* inhibition of *Aspergillus* [14,15,16,17,20]. The physical microscopic correlates of the inhibition, as we have described metabolically, of *Aspergillus* biofilm by these *Pseudomonas* products have recently been documented and detailed by novel observational, computer-driven techniques [46]. Our studies here clearly indicate that virus infection weakens *A. fumigatus* in intermicrobial competition.

The differences demonstrated in susceptibility to *Pseudomonas* were unrelated to any underlying differences in metabolism or growth under the control conditions for the assays. The two reference *Aspergillus* strains, which are infected, despite prolonged storage in, and multiple passes in vitro, in laboratories in different continents, showed negligible differences in susceptibility to *Pseudomonas.* The virus-free *Aspergillus* under those conditions grew slightly, but insignificantly, less well in the absence of *Pseudomonas* than the infected. Had the virus-free grown better than infected, such as might be owing to an impairing effect of infection, one could have considered that the virus-free was more resistant to the predations of *Pseudomonas* merely because it grew better under the study conditions, but the latter was not the case. We would emphasize that the demonstrated equivalence of growth (in the absence of *Pseudomonas*) may not apply to all other growth conditions; other growth conditions would need to be studied individually. Indeed, as yet unpublished studies, with a related virus, in an entomopathogenic fungus, indicate differences between that infected and uninfected fungus, with respect to growth, are medium-dependent [47].

The differential effect of virus on iron-stimulated fungal growth, particularly when coupled with the findings of effects of iron-chelating molecules, such as pyoverdine and PQS, may be a clue that at least some of the resistance differences to *Pseudomonas* products are linked to a virus-induced alteration in *Aspergillus* iron metabolism; iron metabolism is key to *Aspergillus* physiology, as recently reviewed [48]. Subsequent to the findings reported here, in studies in collaboration with other researchers, significant differences in timing of, and amount of, siderophore production between these virus-infected and uninfected isogenic *Aspergillus* strains were discovered, which would explain the strain differences in response to iron-denying *Pseudomonas* products (R. Patil et al., submitted for publication). It is also probable that virus infection weakens *Aspergillus’* ability to respond to any stresses, of which iron denial is one. The best evidence for that hypothesis is the differential susceptibility to *Pseudomonas* volatiles, since small lipophilic organic molecules appear to be the mechanism of such inhibition [18].

## 5. Conclusions

Our novel observations about altered susceptibility of a fungus, *Aspergillus,* to another microbe, *Pseudomonas*, coupled with studies indicating fungal virulence may be depressed by viral infection, and that some viruses may lyse fungi [27,28,29,38], might also have applications in the future for the design of viruses as antifungal treatment [30,31], analogous to phage therapy of bacteria.

## Data Availability

All supplementary materials referred to are available from cimr_admin@cimr.org.

## Supplementary Materials

The following are available online at https://www.mdpi.com/article/10.3390/v13040686/s1. Figure S1: Representative electrophoretic profiles of AF293 infected, virus-free, and re-infected strains. Figure S2: Effect of PAO1 planktonic filtrate on AF293 virus-free or AF293 infected planktonic growth. Supplementary text.
