# Supplementary material for: Virus Infection of Aspergillus fumigatus Compromises the Fungus in Intermicrobial Competition
Source: Viruses. 2021 Apr 16;13(4):686. doi: 10.3390/v13040686 (PMC8073786; doi:10.3390/v13040686)
Supplement: Supplementary file 1 [file viruses-13-00686-s001.zip › viruses-1163524-supplementary.pdf]

Supplementary text

When 2-fold dilutions from 1:2 to 1:256 of *Pseudomonas* supernatant were exposed to *Aspergillus*, growing from conidia, in tubes of liquid media, there was a difference between virus-free and virus-infected *Aspergillus* only at the 1:16 dilution (Supplementary Fig. 1). However, only at this one "breakpoint" dilution were any differences seen between infected and uninfected. At dilutions less than 1:16, all *Aspergillus* strains were completely inhibited, and with greater dilutions, all strains grew equally well. Moreover, when this experiment was repeated 3 times, although the inflection point (1:16 dilution) was the same, in the repeat assays the differences between the *Aspergillus* strains were not statistically significant.

Supplementary figures

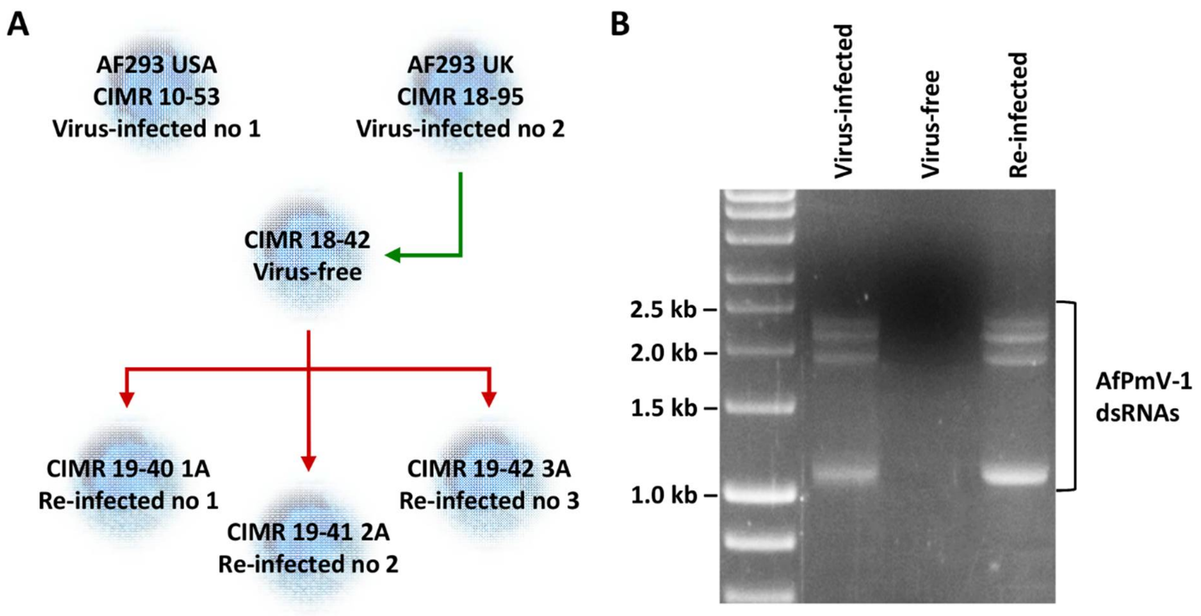

Figure S1. *Representative electrophoretic profiles of AF293 infected, virus-free and re-infected strains.* **A.** Diagram of methods. **B.** The original UK AF293 infected strain was cured of AfPmV-1 using the protein synthesis inhibitor cycloheximide. AfPmV-1 was purified from the infected strain by ultracentrifugation and used to re-infect the AF293 virus-free strain by protoplast transfection, on three occasions. The AfPmV-1 dsRNA genome was extracted from the infected strain and all three re-infected strains and visualized on a 1% (w/v) agarose gel electrophoresis. The reinfected isolates gave identical electrophoretic profiles to the infected isolate in all three such studies. An example is shown. The band sizes of the Hyperladder I are indicated. Adapted from Kanhayuwa L., Kotta-Loizou I., Özkan S., Gunning A.P., Coutts R.H.A. (2015). *Proc. Natl. Acad. Sci. U. S. A.* 112(29):9100-5, with permission.

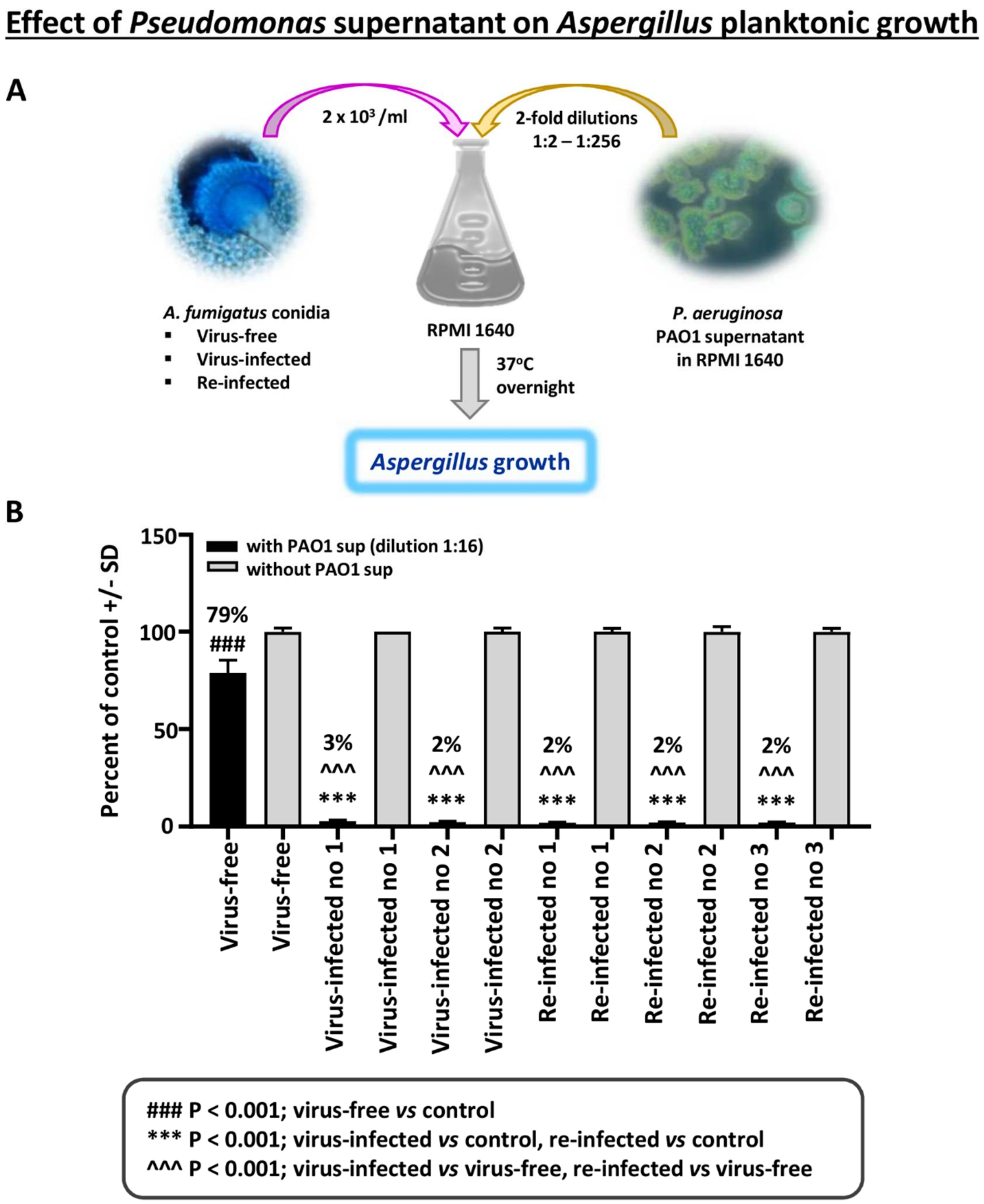

Figure S2. *Effect of PAO1 planktonic filtrate on AF293 virus-free or AF293 infected planktonic growth* Serial twofold dilutions of filtrate were tested in macrodilution, as for an MIC [17]. **A.** Diagram of methods. **B.** At the 1:16 dilution, the virus-free *Aspergillus* was only slightly inhibited (79% compared to control, the *Aspergillus* grown without *Pseudomonas* filtrate), whereas infected strains (both infected strains and all 3 re-infected strains) were still markedly inhibited (2-3% of control). The differences between infected and uninfected were also significant. On a duplicate study, visual inspection of the tubes confirmed these spectrophotometric reading differences. Differences were only seen at this inflection point. At dilutions less than 1:16, all *Aspergillus* strains were completely inhibited, and with greater dilutions, all strains grew equally well.
